# Self-assembled functional components-doped conductive polypyrrole composite hydrogels with enhanced electrochemical performances

**DOI:** 10.1039/d0ra00102c

**Published:** 2020-03-12

**Authors:** Juanjuan Yin, Qingqing Liu, Jingxin Zhou, Lexin Zhang, Qingrui Zhang, Randi Rao, Shufeng Liu, Tifeng Jiao

**Affiliations:** Hebei Key Laboratory of Applied Chemistry, Hebei Key Laboratory of Heavy Metal Deep-Remediation in Water and Resource Reuse, School of Environmental and Chemical Engineering, Yanshan University Qinhuangdao 066004 P. R. China zhangqr@ysu.edu.cn tfjiao@ysu.edu.cn; State Key Laboratory of Metastable Materials Science and Technology, Yanshan University Qinhuangdao 066004 P. R. China; Key Laboratory of Optic-electric Sensing and Analytical Chemistry for Life Science, Ministry of Education, College of Chemistry and Molecular Engineering, Qingdao University of Science and Technology 53 Zhengzhou Road Qingdao 266042 P. R. China

## Abstract

A conductive hydrogel is a composite conductive material formed by combining a conductive polymer with a nanogel structure of a hydrogel. Conductive hydrogels not only have potential applications in supercapacitors, sensors, and modulators, they can also be synthesized by many methods, such as copolymerization, crosslinking, and grafting. In this work, we successfully prepared three conductive composite hydrogels by *in situ* polymerization, namely polypyrrole sodium alginate conductive hydrogel, ferric chloride-doped polypyrrole sodium alginate hydrogel and doped polypyrrole sodium alginate hydrogel with sodium dodecylbenzene sulfonate. In addition, a series of characterizations were performed for the three conductive hydrogels described above. The results show that the polypyrrole sodium alginate hydrogel doped with ferric chloride forms a nanofiber network with a more stable structure and better electrochemical performance.

## Abstract 2

New functional components-doped conductive polypyrrole composite hydrogels are prepared *via* a self-assembled process, demonstrating potential applications in catalysis as well as electrochemical materials.

## Graphical abstract

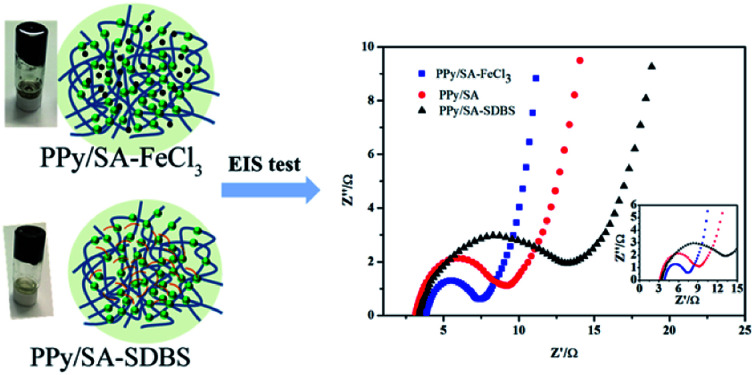

## 1 Introduction

In recent years, many new materials have been developed due to resource shortages. For example, conductive polymer hydrogels are considered ideal electrode materials. Ordered three-dimensional nanostructures have the advantages of a large specific surface area and fast electron/ion transfer.^1^ In particular, nanocomposite hydrogels combine the advantages of hydrogels and nanomaterials, and have outstanding applications in many fields such as sensors, biomimetic materials, biomedicine and pharmaceutical science. The conductivity and mechanical properties of composite hydrogels mainly depend on the structure and distribution of conductive components. Ni *et al.* prepared an efficient and recyclable catalyst by intelligently constructing a palladium@polypyrrole nanocomposite coating on a magnetic carrier.^2^ Deng *et al.* prepared a stimulus-responsive conductive nanocomposite hydrogel with high stretchability, good self-healing, high adhesion and 3D printability, which could be used for human motion sensing.^3^ Polypyrrole (PPy) polymers have excellent electrical conductivity due to the presence of conjugated double bonds. Its conductivity is related to doped electron donors or electron acceptors. She *et al.* prepared a polypyrrole/graphene oxide high-performance swelling hydrogel with a porous layered structure by a one-step self-assembly method.^4^

The presence of sodium alginate makes this molecule useful as a polymer template to alter the aggregation state, morphology, and particle size of polypyrrole. Sodium metal ions in sodium alginate can undergo ion exchange reactions with some heavy metal ions, thereby complexing. Therefore, they have a certain ability to adsorb heavy metal ions and can be used as an adsorbent material for heavy metal ions. Sodium alginate can be combined with divalent or polyvalent cations other than magnesium ions.^5–8^ Considering many advantages of sodium alginate, sodium alginate molecule has a large number of carboxyl and hydroxyl structures. It can be used as an ideal biopolymer for ordered nanostructure template synthesis. Huang *et al.* studied the synthesis of three-dimensional nanostructured polypyrrole/alginate supercapacitor conductive hydrogels by self-assembly.^9^

In addition, sodium dodecylbenzene sulfonate is an anionic surfactant and dopant. Due to its large specific surface area and high conductivity, it plays an important role in the morphology and structure of PPy/SA.^10–17^ Therefore, in this work, a polypyrrole sodium alginate composite conductive hydrogel was synthesized by *in situ* polymerization, and was doped with ferric chloride and sodium dodecylbenzene sulfonate. Finally, the morphologies of the three hydrogels were characterized, and the electrochemical performance of the composite conductive hydrogels was tested by cyclic voltammetry and electrochemical impedance spectroscopy.

## 2 Materials and methods

### 2.1 Materials

Pyrrole (chemically pure) and ammonium persulfate (AR 98%) were purchased from China Aladdin Industrial Co., Ltd., and used after distillation. Sodium alginate (SA, chemically pure) and FeCl_3_ were purchased from Shanghai chemical corporation. Sodium dodecyl benzenesulfonate was purchased from the gangue fine chemical research institute in Tianjin and was not further purified when used. The water used for the experiment is the ultra-pure water and the secondary purified ultra-pure water.

### 2.2 Preparation of polypyrrole hydrogel

At room temperature, 30 μL pyrrole monomer solutions was taken to a small glass bottle with a pipette, and then 35 mg ammonium persulfate was weighed in an analytical balance and dissolved in 400 μL ultra-pure water. The pyrrole hydrogel was then formed in a small glass bottle containing pyrrole monomer solution in ice water. The initiator ammonium persulfate solution was slowly added into the glass bottle and polymerized in an ice water bath.

### 2.3 Preparation of polypyrrole sodium alginate hydrogel

25 mg sodium alginate was put into a small glass bottle at room temperature, and then added with 3 mL ultra-pure water, and conduct magnetic stirring to make the sodium alginate solution dissolve evenly. Next, place the vials in ice water and stir in 30 μL pyrrole monomer for another hour. Finally, 35 mg ammonium persulfate dissolved in 400 μL ultra-pure water was slowly added to a glass vial, polymerized in ice water and allowed to stand for some time to form a stable polypyrrole sodium alginate hydrogel.

### 2.4 Preparation of ferric chloride-doped composite hydrogel

At room temperature, 25 mg sodium alginate was weighted by an analytical balance and put into a glass vial. Then 3 mL ultra-pure water was added to the glass vial for magnetic stirring. Next, place the vials in ice water and stir with 30 μL pyrrole monomer for another hour. Finally, 35 mg ammonium persulfate and 20 mg ferric chloride were dissolved together in 400 μL ultra-pure water and stirred evenly. Then, they were added slowly in ice water and stirred evenly to polymerize in ice water. After a period of time, stable ferric chloride doped polypyrrole sodium alginate hydrogel was formed.

### 2.5 Preparation of sodium dodecyl benzene sulfonate-doped composite hydrogel

At room temperature, 25 mg sodium alginate was weighed by an analytical balance and put into a glass vial. Then 3 mL ultra-pure water was added to the glass vial for magnetic stirring. Next, place the vials in ice water and stir with 30 μL pyrrole monomer for another hour. Finally, 35 mg ammonium persulfate and 20 mg sodium dodecyl benzene sulfonate were dissolved together in 400 μL ultra-pure water and stirred evenly. Then, they were added slowly in ice water and stirred evenly to polymerize in ice water. After a period of time, stable ferric chloride doped polypyrrole sodium alginate hydrogel was formed.

### 2.6 Characterization

X-ray powder diffraction (XRD) pattern was carried out on a D/max-2500/PC X-ray diffractometer with Cu Kα radiation (*λ* = 0.15418 nm). Field emission scanning electron microscopy (FE-SEM) is a testing method for analyzing the physical and microscopic morphology of materials. The SEM equipment used is SUPRA-55 manufactured by Carl Zeiss Company of Germany. The test condition is conventional high vacuum imaging. The range of acceleration voltage is 10–25 kV. Transmission electron microscopy (TEM) is also an important way to analyze the micro-physical morphology of materials. In this work, the instrument model used is HT-7700 transmission electron microscope of Hitachi Company of Japan. The morphology and crystallinity of the particles are observed under 100 kV electrons beam. Before the test, a small amount of samples are grinded and dissolved in ethanol. After ultrasonic dispersion, the upper suspension droplets are placed on the copper net. The morphology and structure of the particles are observed under 100 kV electrons beam. Thermogravimetric (TG) test is an analytical method used to analyze the thermal stability and composition of materials. The model of thermogravimetric analyzer used in this paper is DTG-60 automatic thermogravimetric analyzer of Shimadzu Company, Japan. Temperature range is 0–800 °C, heating rate is 10 °C min^−1^. The used infrared spectroscopy instrument is the Fourier Infrared Spectrometer of Nicolet IS10 Instrument Company, USA. The average scanning times are 32 with the testing wavelength ranges from 4000 to 400 cm^−1^. For testing of electrochemical properties of materials, three-electrode system was used to test the electrochemical performance of the composite hydrogel material. The reference electrode was Ag/AgCl electrode and the electrolyte was 1 M KCl. The cyclic voltammetry (CV) and electrochemical impedance spectroscopy (EIS) were tested by CHI660E electrochemical workstation.

## 3 Results and discussion

### 3.1 Characterization of composite hydrogels

**Fig. 1:**
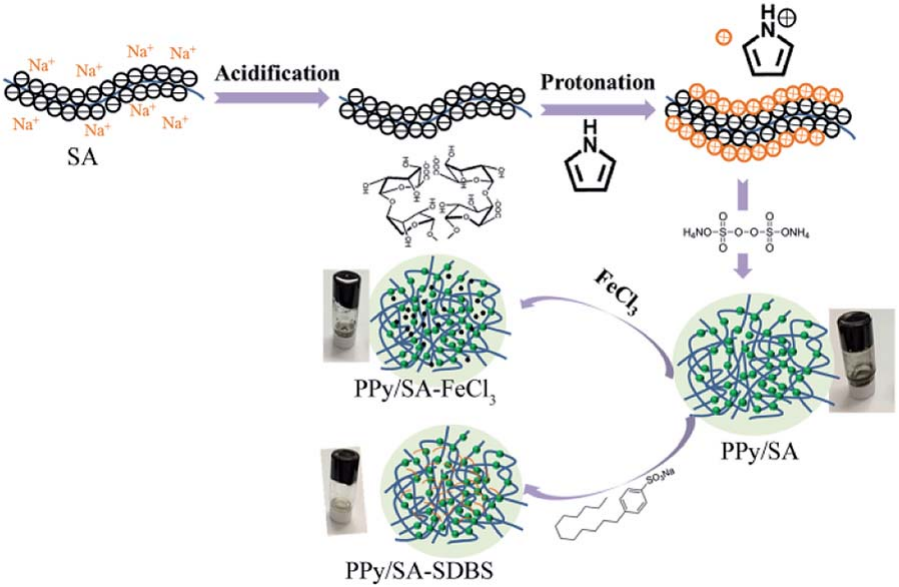
Schematic diagram of synthesis process of composite hydrogels.

Polypyrrole alginate hydrogel was prepared by *in situ* oxidative polymerization. Sodium alginate molecules contain a large number of negatively charged carboxyl and hydroxyl groups. SA can be used not only as a polyelectrolyte with environmental responsiveness, but also as an electrode material in the presence of conductive polymers. Due to the two types of interactions in the system, the prepared conductive hydrogel has a typical 3D nanostructure. One of them is that the entanglement of PPy and SA molecular chains is caused by the interaction of hydrogen bonds (and static electricity). The other is a hydrogen bond in the intermolecular/intramolecular SA chain. The synergy of the two interactions leads to the synthesis of a conductive hydrogel, as shown in Fig. 1.

**Fig. 2:**
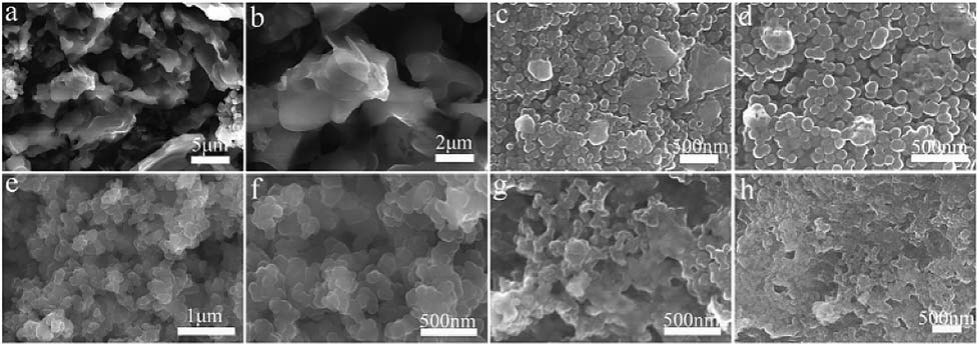
SEM images of composite hydrogels: (a and b) polypyrrole hydrogel; (c and d) polypyrrole sodium alginate hydrogel; (e and f) ferric chloride-doped composite hydrogel; (g and h) sodium dodecyl benzenesulfonate-doped hydrogel.

**Fig. 3:**
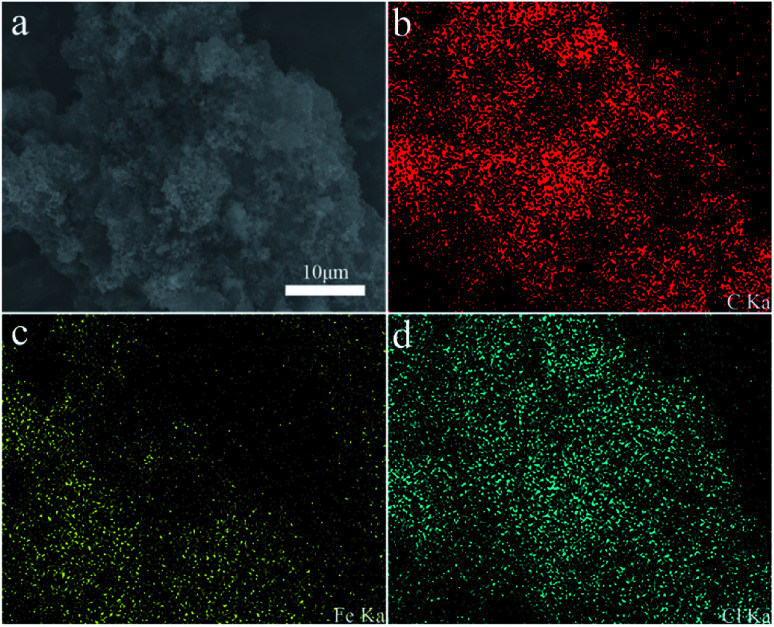
Elemental mapping of ferric chloride-doped composite hydrogel.

The morphology of polypyrrole hydrogel, polypyrrole sodium alginate hydrogel, polypyrrole sodium alginate hydrogel doped with ferric chloride and polypyrrole sodium alginate doped with sodium dodecyl benzene sulfonate were observed by SEM, as shown in Fig. 2. From the scanning image, we can see that the shape of pure polypyrrole is aggregated into blocks, stacked together, showing irregular shape. This is mainly because the polymerization process of pyrrole is a multi-layer growth process.^18^ The polypyrrole polymer is columnar in structure, and above is the nanoscale shape formed by the entanglement and connection of porous three-dimensional nano-network structure.^19^ So the surface of the polypyrrole sodium alginate composite hydrogel we observed from the scanning electron microscope is very rough. Polypyrrole sodium alginate composite hydrogel is a typical nanostructure with a diameter of about 150 nm. At low magnification, scanning electron microscopy (SEM) showed that polypyrrole sodium alginate composite hydrogel is a densely packed dispersed spherical structure. In addition, because sodium alginate was used as a structure directing agent during the reaction, a polypyrrole/sodium alginate (PPy/SA) nanostructure was prepared by *in situ* oxidation polymerization of the pyrrole method. Therefore, by scanning electron microscopy, we can see that polypyrrole sodium alginate composite hydrogel is a kind of three-dimensional nano-structure porous high specific surface area composite hydrogel, which is an ideal material with great potential as the electrode material for supercapacitors. SEM-EDS analyses of polypyrrole sodium alginate hydrogels doped with ferric chloride were shown in Fig. 3. There are uniform and regular dense dots in the cross section, which indicates that Fe^3+^ ions are uniformly distributed and that ferric chloride is doped in the composite.

**Fig. 4:**
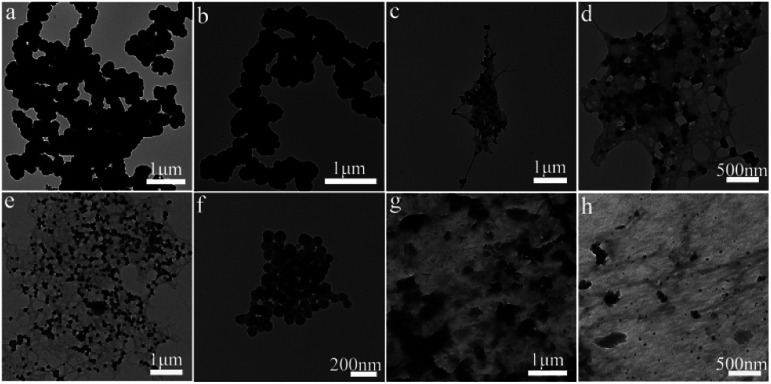
TEM images of composite hydrogels: (a and b) polypyrrole hydrogel; (c and d) polypyrrole sodium alginate hydrogel; (e and f) ferric chloride-doped composite hydrogel; (g and h) sodium dodecyl benzenesulfonate-doped hydrogel.

In order to further study the morphological characteristics of the composite hydrogel, a TEM analysis test was performed on the composite hydrogel, as shown in Fig. 4. We can see that the shape of pure polypyrrole particles is irregular and aggregates into clumps with a very rough surface due to highly oriented molecular interactions (such as hydrogen bonding), which helps to form organized bands or fiber structure. Polypyrrole sodium alginate composite hydrogels exhibit uniform nanostructures, and the consistent morphological characteristics of the nanostructures conform to the morphological characteristics, which have been reported in the literature.^20^ In addition, we observed that the polypyrrole sodium alginate composite hydrogel was doped with ferric chloride and sodium dodecylbenzene sulfonate. It can be seen from the figure that ferric chloride and sodium dodecylbenzene sulfonate have been mixed. The polypyrrole sodium alginate composite hydrogel was successfully doped, and the distribution was more uniform. The results of these tests are consistent with those of scanning electron microscope (SEM-EDS) images.

**Fig. 5:**
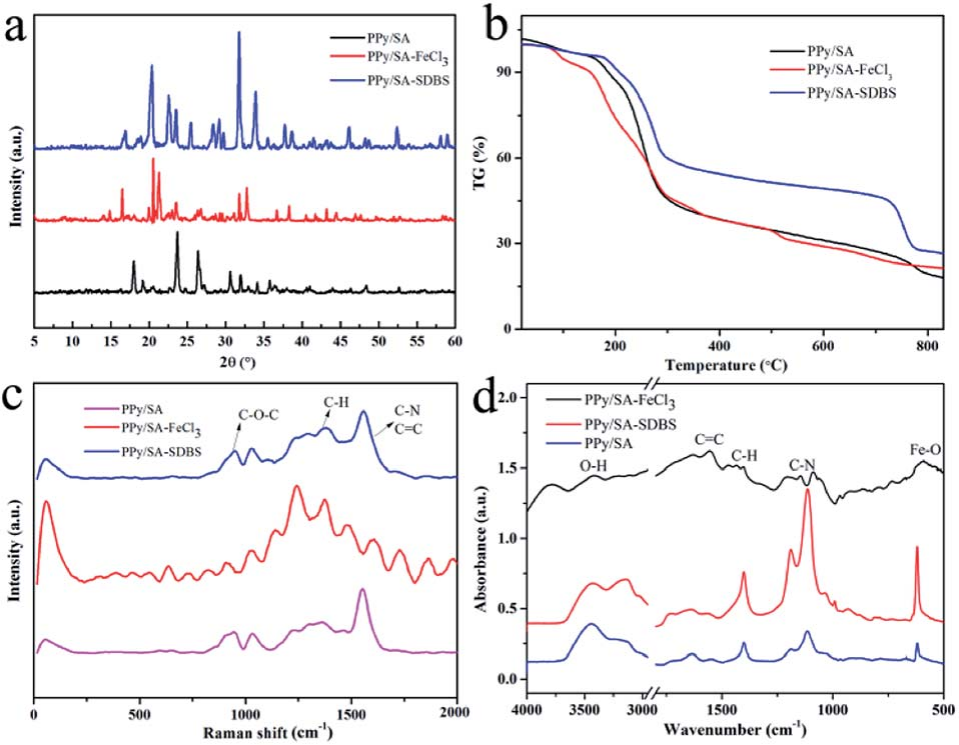
XRD patterns (a), TG curves (b), Raman curves (c), and FT-IR curves (d) of three polypyrrole alginate composite hydrogels.

Through the characterization and analysis of the X-ray diffraction spectra of the composite hydrogel samples, as shown in Fig. 5a, As shown in Fig. 5a, as reported in the literature, a characteristic broad peak of polypyrrole can be seen at 22.8°, which is attributed to the π–π accumulation between the polypyrrole units, indicating that the polypyrrole formed by *in situ* polymerization has a more orderly arrangement and the structural defects are significantly reduced.^21^ At 2*θ* = 27° mainly depends on the height of dopant, key conditions of supramolecular hydrogel is that hydrogen bonds exist in the hydrogel. There was no obvious diffraction peak in the polypyrrole sodium alginate composite hydrogel, which indicated that the content of sodium alginate in the polypyrrole sodium alginate composite hydrogel was very low, and the alginate was uniformly dispersed in the composite hydrogel. In addition, the diffraction angle of composite hydrogel by 2*θ* = 25° near migrated to near 2*θ* = 31°, what can be inferred according to the change, ferric chloride and sodium dodecyl benzene sulfonic acid doped into the polypyrrole sodium alginate composite hydrogel, the composite hydrogel formed we expected.

TG is a test used to study the thermal stability and composition of composite hydrogels.^22–25^ In order to further study the thermal stability of composite hydrogels,^26^ thermogravimetric analyses was used. As shown in Fig. 5b, the thermogravimetric curves of polypyrrole sodium alginate compound hydrogels doped with ferric chloride and sodium dodecylbenzene sulfonic acid in the atmosphere of nitrogen are shown respectively. According to the analysis in the figure, these three compound hydrogels show basically similar weight change behaviors. Before the temperature of polypyrrole sodium alginate composite hydrogel increased to 150 °C, the weight of the composite hydrogel decreased, but the decrease was slower. This may be due to the evaporation of some water during the temperature rise. Between 150–290 °C, the weight sending changes dramatically. According to similar reports, this is because the oxygen-containing functional groups and organic groups of the polypyrrole sodium alginate composite hydrogel undergo thermal decomposition reactions.^27–33^ For doped polypyrrole of dodecyl benzene sulfonic acid sodium alginate composite hydrogel, between 150–290 °C weightlessness slower, it illustrates the addition of sodium dodecyl benzene sulfonic acid increased the polypyrrole sodium alginate composite hydrogel between thermal stability, at the same time, the polypyrrole chains with the combination of the chemical bonds between dodecyl benzene sulfonic acid sodium and also further improve the interaction between polypyrrole thermal stability of sodium alginate composite hydrogel. It is interesting to note that the thermogravimetric curve reaches a plateau at 290 °C, where all the quality of the composite hydrogel remains relatively stable. The obtained data indicated the prepared composite hydrogel had good thermal stability. The molecular backbone can be determined from Raman spectroscopy, as shown in Fig. 5c. The peaks at 950 cm^−1^, 1374 cm^−1^ and 1566 cm^−1^ correspond to the molecular frameworks of –C–O–C–, –C–H and –C=C–, respectively. In addition, polypyrrole sodium alginate and doped polypyrrole of ferric chloride sodium alginate composite hydrogel almost quality loss, and doped with dodecyl benzene sulfonic acid sodium polypyrrole sodium alginate composite hydrogel quality loss is relatively less, mainly because the composite hydrogel samples are difficult to decompose due to the material.

Three kinds of hydrogels were characterized by infrared spectroscopy. Fourier transform infrared spectroscopy used molecular vibration to obtain the spectrum.^34–38^ Infrared absorption spectrum can detect functional groups in materials, as shown in Fig. 5d. The peaks of 1628 cm^−1^ and 1416 cm^−1^ are –COO^−^ asymmetric tensile vibration and –COO^−^ symmetric tensile vibration. The peak values of 1308 cm^−1^ and 1043 cm^−1^ were C–H plane deformation. The peaks of 1176 cm^−1^ and 898 cm^−1^ were external tensile vibrations. With ferric chloride of polypyrrole sodium alginate hydrogel with Fe–O features keys in 576 cm^−1^, doped polypyrrole of ferric chloride sodium alginate C=C double bond, is due to the pyrrole ring conjugation effect of C=C, anionic surfactant with polypyrrole anion electrostatic interaction can be doped into polyethylene pyrrole polymer to form a composite structure, and doped polypyrrole of dodecyl benzene sulfonic acid sodium, sodium alginate hydrogels have characteristic peak of dodecyl benzene sulfonic acid sodium. A wider peak appears at 1160–1180 cm^−1^ in sodium dodecyl benzenesulfonate, indicating the interaction of sulfonate group with N of sulfonamide.^39–46^

### 3.2 Electrochemical properties of composite hydrogels

**Fig. 6:**
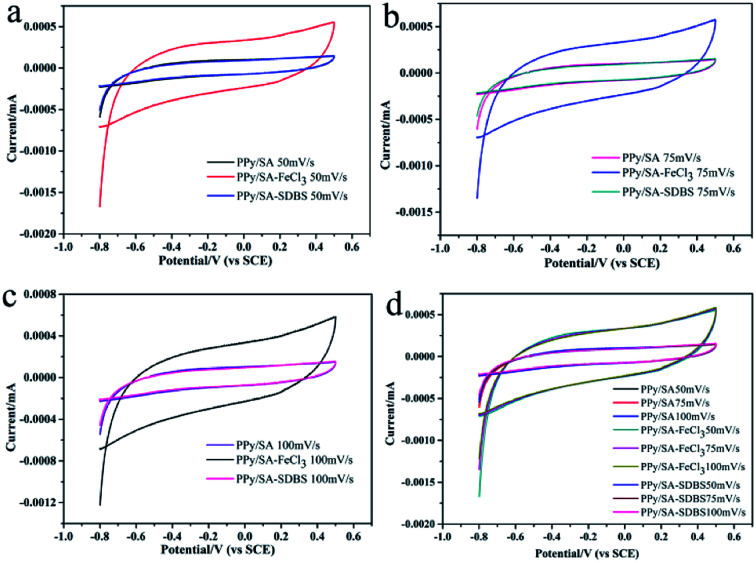
Cyclic voltammograms of three polypyrrole sodium alginate composite hydrogels at scanning speeds of 50 mV s^−1^ (a), 75 mV s^−1^ (b), 100 mV s^−1^ (c), and total comparison (d).

Three kinds of composite hydrogels were tested by cyclic voltammetry, as shown in Fig. 6, it can be seen that the hydrogels have pseudo capacitance, and the cyclic voltammetry curve is close to the rectangle, indicating that there is charge movement in the composite hydrogels. And the cyclic voltammetry characteristic curve is close to the rectangle with good cyclic stability. When the scanning speed is slow, the ions in the electrolyte have enough time to diffuse to each position inside the electrode and react fully with polypyrrole. On the contrary, when the scanning rate is very fast, the ions in the electrolyte do not have sufficient time, so they cannot be fully moved to the inside of the electrode. In addition, according to Fig. 6a–c, it can be concluded that the area enclosed by the cycle closure curve of PPy/SA-FeCl_3_ is larger than the area enclosed by the cycle closure curve of PPy/SA and PPy/SA-SDBS at the same sweep speed, which shows that PPy/SA-FeCl_3_ has a higher specific capacity.

**Fig. 7:**
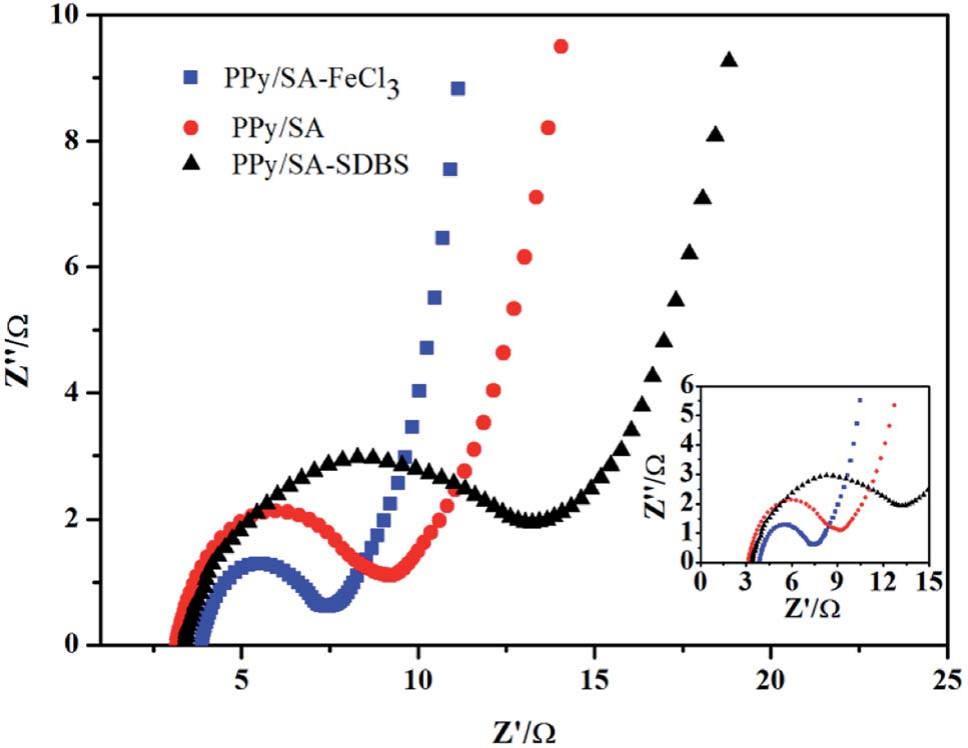
Electrochemical impedance spectra of three polypyrrole sodium alginate composite hydrogels.

Electrochemical impedance tests were performed to study the kinetic properties of composite hydrogels. As shown in Fig. 7, the electrochemical impedance spectra of the electrode material polypyrrole sodium alginate composite hydrogel doped with ferric chloride and dodecyl benzene sulfonate were shown respectively. It can be seen from the figure that under the high-frequency arc radius, the polypyrrole of the doped sodium trichloride alginate composite hydrogel is smaller than that of the doped sodium dodecylbenzenesulfonate composite hydrogel. It shows that the doped ferric trichloride sodium alginate composite hydrogel has a small charge transfer resistance, and the surface charge transfer resistance is smaller than that of the dodecylbenzenesulfonic acid doped sodium alginate composite hydrogel. Therefore, it is more conducive to charging and also improves its electrochemical performance. This is because the polypyrrole sodium alginate hydrogel can be doped with ferric chloride to form a uniform fiber structure (shown in Fig. 2e and f), resulting in a small charge transfer resistance and an internal channel for charge transfer. At the same time, the slope of the low-frequency polypyrrole sodium alginate composite hydrogel doped with ferric chloride was slightly higher than that of the polypyrrole sodium alginate composite hydrogel doped with dodecyl benzene sulfonate, indicating that the polypyrrole sodium alginate composite hydrogel doped with ferric chloride had good double-capacitance properties. The electrochemical dynamic properties of the polypyrrole alginate hydrogel doped with ferric chloride were better than those of the polypyrrole alginate hydrogel doped with SDBS.

## 4 Conclusions

In summary, a polypyrrole alginate conductive composite hydrogel was prepared by *in situ* polymerization, and then ferric chloride and sodium dodecylbenzene sulfonate were doped into the polypyrrole alginate conductive composite hydrogel. The morphology and properties of polypyrrole sodium alginate hydrogel and doped composite hydrogels were characterized by a series of characterization methods, and the gels were tested for charge and discharge tests and electrochemical impedance tests. The obtained results show that the polypyrrole alginate hydrogel doped with ferric chloride has typical pseudo-capacitance characteristics, good charge–discharge rate performance, and good capacitance performance, which is due to polypyrrole seaweed doped with ferric chloride sodium hydrogels with relatively stable nanostructures. Therefore, the electrochemical kinetics of polypyrrole sodium alginate doped with ferric chloride is superior to hydrogel doped with sodium dodecylbenzenesulfonate. Therefore, this work provides new ideas for the design and preparation of nano-conductive composite hydrogels.

## Conflicts of interest

There are no conflicts to declare.

## Supplementary Material

## References

[cit1] Koetting M., Peters J., Steichen S., Peppas N. (2015). Mater. Sci. Eng..

[cit2] Ni X., Qiu J., Li Y., Zhao Y., Yang C., Hong L. (2018). New J. Chem..

[cit3] Deng Z., Hu T., Lei Q., He J., Ma P., Guo B. (2019). ACS Appl. Mater. Interfaces.

[cit4] She X., Sun P., Yu X., Zhang Q., Wu Y., Li L., Jiang S. (2014). J. Inorg. Organomet. Polym. Mater..

[cit5] Fang L., Zhao L., Liang X., Xiao H., Qian L. (2016). J. Appl. Polym. Sci..

[cit6] Wang Y., Shi Y., Pan L., Ding Y., Zhao Y., Li Y., Yu G. (2015). Nano Lett..

[cit7] Yang S., Jang L., Kim S., Yang J., Yang K., Cho S., Lee J. (2016). Macromol. Biosci..

[cit8] Li S., Liu J., Zhang X., Li L., Yu X., Huang Z. (2015). Polym. Bull..

[cit9] Huang H., Yao J., Liu Y., Tuo X., Da Y., Zeng X., Li L. (2017). J. Macromol. Sci..

[cit10] Pattananuwat P., Aht-ong D. (2017). Electrochim. Acta.

[cit11] Guo H., Jiao T., Shen X., Zhang Q., Li A., Zhou J., Gao F. (2014). Colloids Surf., A.

[cit12] Zhao H., Jiao T., Zhang L., Zhou J., Zhang Q., Peng Q., Yan X. (2015). Sci. China Mater..

[cit13] Zhu K., Jiao T., Zhang L., Xing R., Guo R., Zhou J., Li X. (2016). Sci. Adv. Mater..

[cit14] Svobodová H., Noponen V., Kolehmainen E., Sievänen E. (2012). RSC Adv..

[cit15] Liang X., Qu B., Li J., Xiao H., He B., Qian L. (2015). React. Funct. Polym..

[cit16] Zheng J., Yu X., Wang C., Cao Z., Yang H., Ma D., Xu X. (2016). J. Mater. Sci..

[cit17] Zhang L., Zheng J., Dou P., Wang W., Cheng J., Xu X. (2017). J. Mater. Sci..

[cit18] Krishnappa R., Desai K., Sung C. (2003). J. Mater. Sci..

[cit19] Li Y., Zhao X., Xu Q., Zhang Q., Chen D. (2011). Langmuir.

[cit20] Kessick R., Tepper G. (2004). Appl. Phys. Lett..

[cit21] Wang Y., Shi Y., Pan L., Ding Y., Zhao Y., Li Y., Shi Y., Yu G. (2015). Nano Lett..

[cit22] Li H., Yin J., Meng Y., Liu S., Jiao T. (2020). Colloids Surf., A.

[cit23] Meng Y., Yin J., Jiao T., Bai J., Zhang L., Su J., Liu S., Bai Z., Cao M., Peng Q. (2020). J. Mol. Liq..

[cit24] Song J., Yuan C., Jiao T., Xing R., Yang M., Adams D. J., Yan X. (2020). Small.

[cit25] Feng Y., Yin J., Liu S., Wang Y., Li B., Jiao T. (2020). ACS Omega.

[cit26] Guo H., Jiao T., Zhang Q., Guo W., Peng Q., Yan X. (2015). Nanoscale Res. Lett..

[cit27] Bu Y., Xu H., Li X., Xu W., Yin Y., Dai H., Xu P. (2018). RSC Adv..

[cit28] Hou N., Wang R., Geng R., Wang F., Jiao T., Zhang L., Zhou J., Bai Z., Peng Q. (2019). Soft Matter.

[cit29] Zhu J., Wang R., Geng R., Zhang X., Wang F., Jiao T., Yang J., Bai Z., Peng Q. (2019). RSC Adv..

[cit30] He Y., Wang R., Jiao T., Yan X., Wang M., Zhang L., Bai Z., Zhang Q., Peng Q. (2019). ACS Sustain. Chem. Eng..

[cit31] Mit-uppatham C., Nithitanakul M., Supaphol P. (2004). Macromol. Chem. Phys..

[cit32] Megelski S., Stephens J., Chase D., Rabolt J. (2002). Macromolecules.

[cit33] Demir M., Yilgor I., Yilgor E., Erman B. (2002). Polymer.

[cit34] Ma K., Wang R., Rao Y., Zhao W., Liu S., Jiao T. (2020). Colloids Surf., A.

[cit35] He Y., Wang R., Sun C., Liu S., Zhou J., Zhang L., Jiao T., Peng Q. (2020). ACS Omega.

[cit36] Zhang L., Yin J., Wei K., Li B., Jiao T., Chen Y., Zhou J., Peng Q. (2020). Nanotechnology.

[cit37] Cai C., Wang R., Liu S., Yan X., Zhang L., Wang M., Tong Q., Jiao T. (2020). Colloids Surf., A.

[cit38] Xu Y., Wang R., Zheng Y., Zhang L., Jiao T., Peng Q., Liu Z. (2020). Appl. Surf. Sci..

[cit39] Geng R., Yin J., Zhou J., Jiao T., Feng Y., Zhang L., Chen Y., Bai Z., Peng Q. (2020). Nanomaterials.

[cit40] Zhan F., Yin J., Zhou J., Jiao T., Zhang L., Xia M., Bai Z., Peng Q. (2020). Nanomaterials.

[cit41] Feng Y., Wang R., Yin J., Zhan F., Chen K., Jiao T., Zhou J., Zhang L., Peng Q. (2020). Curr. Nanosci..

[cit42] Zhao J., Yin J., Zhong J., Jiao T., Bai Z., Wang S., Zhang L., Peng Q. (2020). Nanotechnology.

[cit43] Yin J., Zhan F., Jiao T., Deng H., Zou G., Bai Z., Zhang Q., Peng Q. (2020). Chin. Chem. Lett..

[cit44] Ma K., Wang R., Jiao T., Zhou J., Zhang L., Li J., Bai Z., Peng Q. (2020). Colloids Surf., A.

[cit45] Wang C., Yin J., Han S., Jiao T., Bai Z., Zhou J., Zhang L., Peng Q. (2019). Catalysts.

[cit46] Ma K., Chen W., Jiao T., Jin X., Sang Y., Yang D., Zhou J., Liu M., Duan P. (2019). Chem. Sci..

